# Formulation and Evaluation of Tramadol hydrochloride Rectal Suppositories

**DOI:** 10.4103/0250-474X.45405

**Published:** 2008

**Authors:** M. A. Saleem, M. Taher, S. Sanaullah, M. Najmuddin, Javed Ali, S. Humaira, S. Roshan

**Affiliations:** Luqman College of Pharmacy, Old Jewargi road, Gulbarga-585 102, India; 1 Dept. of Pharmaceutics, Faculty of Pharmacy, Hamdard University, New Delhi-110 062, India

**Keywords:** Agar, cocoa butter, *in vitro* release, liquefaction, macromelting, PEG, tramadol hydrochloride

## Abstract

Rectal suppositories of tramadol hydrochloride were prepared using different bases and polymers like PEG, cocoa butter, agar and the effect of different additives on *in vitro* release of tramadol hydrochloride was studied. The agar-based suppositories were non-disintegrating/non-dissolving, whereas PEGs were disintegrating/dissolving and cocoa butter were melting suppositories. All the prepared suppositories were evaluated for various physical parameters like weight variation, drug content and hardness. The PEG and cocoa butter suppositories were evaluated for macromelting range, disintegration and liquefaction time. *In vitro* release study was performed by USP type I apparatus. The prepared suppositories were within the permissible range of all physical parameters. *In vitro* drug release was in the order of PEG>Agar>cocoa butter. Addition of PVP, HPMC in agar suppositories retards the release. The mechanism of drug release was diffusion controlled and follows first order kinetics. The results suggested that blends of PEG of low molecular weight (1000) with high molecular weight (4000 and 6000) in different percentage and agar in 10% w/w as base used to formulate rapid release suppositories. The sustained release suppositories can be prepared by addition of PVP, HPMC in agar-based suppositories and by use of cocoa butter as base.

Rectal drug delivery has a number of advantages such as reduced hepatic first pass elimination of high clearance drugs, avoidance of gastric irritation associated with certain drugs in case of nausea, vomiting and when the patient is unconscious. Rectal route of administration is specifically useful for infants and children who have difficulty in swallowing oral medicine. Drug administered in suppository form can produce not only local effect but also systemic therapeutic action^1^. Suppositories can be prepared by using lipophilic bases like cocoa butter or by hydrophilic bases such as PEGs^2^–^4^. These suppositories melt or dissolve in body fluids and release the drug, but are unstable at higher temperature. Agar has been recently used as base to produce non-disintegrating/non-dissolving suppositories, which are stable at higher temperature^5^,^6^. Tramadol hydrochloride is a synthetic opioid analgesic used for moderate to severe pain like labor pain, traumatic pain, postoperative surgical pain and cancer pain. Tramadol hydrochloride can be administered orally, intravenously or rectally^7^. Tramadol hydrochloride is rapidly absorbed orally but is subjected to first pass metabolism and only 68% is bioavailable after a single oral dose. Common side effects of tramadol hydrochloride include nausea, vomiting, dryness of mouth and sedation^8^. In the present study attempts were made to formulate rectal suppositories of tramadol hydrochloride with different bases like lipophilic base cocoa butter and hydrophilic bases PEGs and agar, as the rectal route avoids first pass metabolism and side effects.

Tramadol hydrochloride was gift sample from Virupaksha Organics Pvt Ltd., Medak, India. PEG 1000 was obtained from Hi-Media Pvt. Ltd., Mumbai. PEG 4000 and PEG 6000 were purchased from Loba Chemie Pvt. Ltd., Mumbai. HPMC, PVP, propylene glycol and bees wax were purchased from S. D. Fine Chemicals Pvt. Ltd., Mumbai, and cocoa butter from Genuine Chemicals, Mumbai. All other chemicals used were of analytical grade.

Agar suppositories were prepared by molding method^9^, dissolving methyl and propyl paraben in hot water and then drug along with other additives like propylene glycol, HPMC, PVP was added and mixed well. Finally agar was incorporated by maintaining the temperature at 75-80° and mixed thoroughly. The molten mass was poured into previously calibrated stainless steel mould of 1g and allowed to set. The PEG suppositories were prepared by fusion method^9^ by melting PEG (1000, 4000 and 6000) in different ratios and then drug was dispersed. Cocoa butter suppositories were prepared by melting cocoa butter and bees wax on water bath, and then the drug was incorporated. The details of all formulations are tabulated in Table 1. All the prepared suppositories were packed in polyethylene laminated foil pouches.

**TABLE 1:** FORMULATIONS OF TRAMADOL HYDROCHLORIDE RECTAL SUPPOSITORIES

Ingredients (%w/w)	Formulation codes												
	
	A0	A1	A2	A3	A4	A5	P1	P2	P3	P4	C1	C2	C3
Tramadol hydrochloride	5	5	5	5	5	5	5	5	5	5	5	5	5
Agar	10	10	10	10	10	10	--	--	--	--	--	--	--
Propylene glycol	--	10	10	10	10	10	--	--	--	--	--	--	--
Methyl paraben	0.03	0.03	0.03	0.03	0.03	0.03	--	--	--	--	--	--	--
Propyl paraben	0.02	0.02	0.02	0.02	0.02	0.02	--	--	--	--	--	--	--
HPMC	--	--	1	3	--	--	--	--	--	--	--	--	--
PVP	--	--	--	--	1	3	--	--	--	--	--	--	--
Water qs	100	100	100	100	100	100	--	--	--	--	--	--	--
PEG 4000	--	--	--	--	--	--	20	40	--	--	--	--	--
PEG 6000	--	--	--	--	--	--	--	--	20	40	--	--	--
PEG 1000 qs	--	--	--	--	--	--	80	60	80	60	--	--	--
Bees wax	--	--	--	--	--	--	--	--	--	--	--	1	3
Cocoa butter qs	--	--	--	--	--	--	--	--	--	--	100	100	100

HPMC: Hydroypropolymethylcellulose, PVP: Polyvinylpyrolidine, PEG: Polyethyleneglycol

Prepared suppositories were visually inspected. Randomly selected suppositories were cut longitudinally and the surfaces were examined with naked eye. For determination of weight uniformity, twenty suppositories were weighed individually and the average weights were determined^10^. No suppositories should deviate from average weight by more than 5% except two, which may deviate by not more than 7.5%.

The drug content for agar and PEG suppositories was determined by soaking individual suppository in water for 30 min, broken with spatula, vortexed for 5 min, filtered, diluted to 50 ml with distilled water, then tramadol hydrochloride was estimated by Shimadzu UV/visible spectrophotometer at 271 nm. For cocoa butter suppositories, drug was extracted by heating the suppository in distilled water at 50° for 5 min, shaking the mixture in separating funnel, separated out the aqueous layer, diluted to 50 ml with distilled water and then estimated at 271 nm.

The hardness of the prepared suppositories was tested using Monsanto hardness tester. Hardness test or breaking strength test was carried to determine the tensile strength of the suppositories to access whether they will be able to withstand the hazards of packing and transporting^11^.

USP tablet disintegration apparatus was employed to measure the melting range of PEG and cocoa butter suppositories^12^. The time taken for the entire suppositories to melt/disperse was measured when immersed in water bath maintained at constant temperature of 37±0.5°.

Ease of insertion of suppositories was evaluated in rabbits^5^. The results were represented as − poor, + fare, ++ good for ease of insertion. Liquefaction temperature/time test was done using fabricated instrument^13^. A big pipette was taken having a narrow opening on one side and broad opening on another side. The pipette was dipped in hot water maintained at 35±0.2° so that narrow end faces towards hot water. The sample suppository was introduced from the top of the pipette through broad end and carefully pushed down its length until it reaches narrow end. A glass rod was then inserted so that it rests over the suppository. The temperature at which the glass rods just come down was noted, that represents the liquefaction temperature. The time at which glass rod reaches to narrow end after complete melting of suppositories represents the liquefaction time.

The disintegration time^11^ was recorded utilizing USP tablet disintegration tester containing distilled water at 37 ± 0.5°. For *in vitro* dissolution studies an Electrolab USP XXIII dissolution apparatus was used^14^. The dissolution medium was 900 ml of distilled water, maintained at 37±0.5°. The suppository was placed in the metal basket and maintained at 50 rpm. Ten millilitres of sample was withdrawn at different intervals of time (10, 20, 30, 45, 60, 90, 120, 180, 240 min) and absorbance was measured at 271 nm. The study was performed for 4 h, except for PEG suppositories, which was studied for 30 min.

All the suppositories were free from pits, fissures and cracks. The longitudinal section of the suppositories was plain and clear. The results of different evaluation parameters are shown in Table 2. The weight variation study for all the suppositories were found to be within the acceptable range of <5%, which indicates that calibration of mold was perfect. All the prepared suppositories showed uniformity in drug content and were within the permissible range (97% to 105%) indicating uniformity of drug dispersion in suppositories. The suppositories should have good mechanical strength for handling and transportation. All the suppositories were having good mechanical strength in the range of 1.50 to 4.00 kg/cm^2^ showing optimum hardness. In PEG suppositories, increasing the concentration of PEG 4000 and PEG 6000 increased the mechanical strength. In cocoa butter suppositories, mechanical strength was increased as the amount of bees wax increased up to 3% w/w and beyond this concentration brittle suppositories were formed. The melting range test was not performed for agar-based suppositories, as it did not contain any fatty or waxy materials. In PEG suppositories, the macromelting range time was increased (from 15° to 32°), as the amount of PEG 4000 and 6000 was increased. In cocoa butter suppositories, the melting range time was improved (from 9° to 15°) by increasing the amount of bees wax. Suppository should have proper stiffness to facilitate insertion in the rectum. All the PEG and cocoa butter suppositories were found to have good stiffness and easily inserted into the rectum of the rabbit. In agar suppositories, addition of 10% propylene glycol improves the ease of insertion. The *in vitro* liquefaction time is the time necessary for a suppository to liquefy under pressure similar to those found in the rectum. The test was done for PEG and cocoa butter suppositories. In PEG suppositories the liquefaction time and temperature was increased as the amount of PEG 4000 and PEG 6000 increased. In cocoa butter suppositories, the liquefaction time and temperature was increased as the amount of bees wax was increased. The disintegration test for agar based suppositories results in no disintegration of the suppositories even after 2 h, suggested that it is non-disintegrating suppository. All the PEG suppositories were disintegrated within a time period of 4-9 min, suggesting that PEG itself was a good disintegrant. Disintegration of cocoa butter suppositories showed a loss in shape of the suppository within 4 to 7 min. The *in vitro* release profiles of tramadol hydrochloride from different bases are shown in figs. 1 and 2. The overall release of tramadol hydrochloride from different bases was as follows, PEG>agar>cocoa butter. Dissolution study of agar-based suppositories indicated that the suppository does not disintegrate, melt or dissolve in the dissolution medium but remains intact. The drug diffuses out from the hydrophilic matrix with time. It was observed that more than 50% of the drug was released from A_0_ formulation within 60 min. Addition of 10% w/w propylene glycol accelerates the release of tramadol hydrochloride significantly (*P*<0.05) as in A_1,_ which may be due to decrease in the gel matrix of agar. In formulation A_2_, A_3_ addition of HPMC (1%, 3% w/w) and in formulation A_4_, A_5_ addition of PVP (1%, 3% w/w) retards the release significantly (*P*<0.05), which may be due to increase in the viscosity and gel strength of the polymer matrix. Hence, PVP, HPMC and similar polymers in higher concentration can be used to formulate sustained released suppositories. The drug released from the PEG suppositories as a consequence of the progressive dissolution of PEG in the dissolution media. Suppositories prepared with the combination of PEG (P_1_–P_4_) showed maximum release of 90% within first 30 min. The combination of different PEG did show any significant (*P*>0.05) effect on *in vitro* release of tramadol hydrochloride. In cocoa butter suppositories, the drug release was very slow and only 15% of the drug was released within 4 h. Addition of bees wax (1%, 3% w/w) further decreased the drug release significantly (*P*<0.05), which may be due to increase in the hardness and liquefaction time. Slow release of tramadol hydrochloride from cocoa butter base was due to high lipophylicity of the base, high water solubility of tramadol hydrochloride, non-miscibility of the base with the dissolution media, absence of additives or surface-active agents. A matrix network structure has been formed during the preparation of suppository with highly water-soluble drug and particle remains suspended in highly lipophillic matrix^4^,^15^. The mechanism of drug release was chiefly diffusion controlled following first order release kinetics with their high value of regression coefficients.

**Fig. 1:**
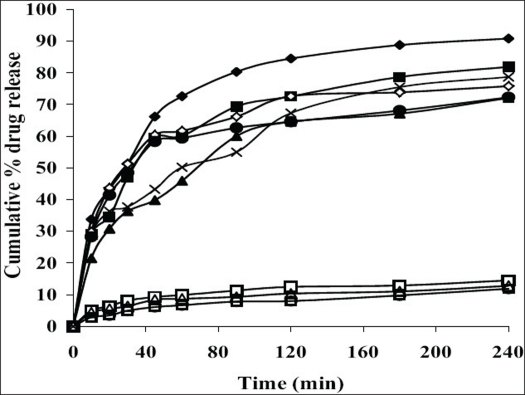
Comparative *in vitro* release of tramadol hydrochloride from different suppositories Comparative *in vitro* release of tramadol hydrochloride from different agar, A0 (–×–), A1 (–♦–), A2 (–■–), A3 (–▲–), A4 (–◊–), A5 (–●–) and cocoa butter, C1 (–□–), C2 (–Δ–), C3 (–○–) suppositories.

**Fig. 2:**
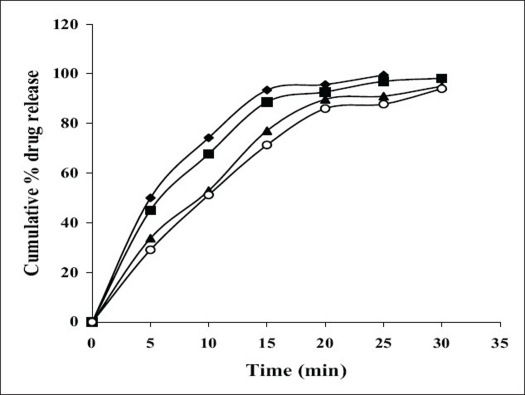
Comparative *in vitro* release of tramadol hydrochloride from different PEG suppositories Comparative *in vitro* release of tramadol hydrochloride from different PEG, P1 (–♦–), P2 (–■–), P3 (–▲–), P4 (–○–) suppositories

**TABLE 2:** EVALUATION OF SUPPOSITORIES FOR VARIOUS PARAMETERS

Formulation Code	Drug content* (%)	Weight variation ±SD (g)	Hardness* (kg/cm^2^)	Disintegration time* (min)	Liquefaction*		Macromelting range* (min)	Ease of insertion

					Time (min)	Temperature (°)		
A0	97.55	1.008±0.04	1.50	--	--	--	--	--
A1	97.00	0.997±0.04	2.00	--	--	--	--	+
A2	98.10	0.998±0.03	2.00	--	--	--	--	+
A3	101.30	1.001±0.01	2.00	--	--	--	--	+
A4	105.00	1.002±0.02	2.00	--	--	--	--	+
A5	99.20	1.004±0.02	2.50	--	--	--	--	+
P1	98.40	1.020±0.01	3.00	4.20	13	38.5	15	++
P2	99.28	1.020±0.01	3.00	5.15	19	41.0	22	++
P3	98.90	0.996±0.01	3.50	8.00	25	40.0	27	++
P4	99.28	1.003±0.02	4.00	9.00	28	43.0	32	++
C1	100.10	0.998±0.02	3.00	4.00	5.30	37.0	9	++
C2	98.70	0.998±0.01	3.00	5.30	6.10	37.5	12	++
C3	98.04	1.002±0.01	3.50	6.15	7.30	38.0	15	++

* Average of three determinants

-- Poor

+ Fair

++ Good

Thus the study suggested that the blends of PEG of low molecular weight (1000) with high molecular weight (4000 and 6000) in different percentage and agar in 10% w/w as base used to formulate conventional rapid release suppositories where as sustained release suppositories can be prepared by addition of PVP, HPMC in agar-based suppositories and by use of cocoa butter as base. Studies conducted so far yielded promising results, thus suggesting a scope for further pharmacokinetic evaluation.
